# Changes in plasma chemokine C-C motif ligand 2 levels during treatment with eicosapentaenoic acid predict outcome in patients undergoing surgery for colorectal cancer liver metastasis

**DOI:** 10.18632/oncotarget.8579

**Published:** 2016-04-04

**Authors:** Milene Volpato, Sarah L Perry, Gemma Marston, Nicola Ingram, Andrew J. Cockbain, Heather Burghel, Mann Jake, David Lowes, Erica Wilson, Alastair Droop, Juliette Randerson-Moor, P Louise Coletta, Mark A Hull

**Affiliations:** ^1^ Leeds Institute of Biomedical & Clinical Sciences, Wellcome Trust Brenner Building, St James's University Hospital, Leeds LS9 7TF, United Kingdom; ^2^ Leeds Institute of Cancer Studies and Pathology, Wellcome Trust Brenner Building, St James's University Hospital, Leeds LS9 7TF, United Kingdom; ^3^ MRC Medical Bioinformatics Centre, University of Leeds, Leeds, LS2 9NL, UK

**Keywords:** colorectal cancer, molecular pharmacology, prognosis, eicosapentaenoic acid and biomarker

## Abstract

The mechanism of the anti-colorectal cancer (CRC) activity of the omega-3 fatty acid eicosapentaenoic acid (EPA) is not understood. We tested the hypothesis that EPA reduces expression of chemokine C-C motif ligand 2 (CCL2), a pro-inflammatory chemokine with known roles in metastasis.

We measured CCL2 in clinical samples from a randomized trial of EPA in patients undergoing liver surgery for CRC liver metastasis (LM) and preclinical models. Genome-wide transcriptional profiling of tumors from EPA-treated patients was performed.

EPA decreased CCL2 synthesis by CRC cells in a dose-dependent manner. CCL2 was localized to malignant epithelial cells in human CRCLM. EPA did not reduce CCL2 content in human or mouse tumors compare to control. However, EPA treatment was associated with decreased plasma CCL2 levels compared with controls (P=0.04). Reduction in plasma CCL2 following EPA treatment predicted improved disease-free survival (HR 0.32; P=0.003). Lack of ‘CCL2 response’ was associated with a specific CRCLM gene expression signature.

In conclusion, reduction in plasma CCL2 in patients with CRCLM treated with EPA predicts better clinical outcome and a specific tumor gene expression profile. Further work is needed to validate CCL2 as a therapeutic response biomarker for omega-3 fatty acid treatment of CRC patients.

## INTRODUCTION

Approximately one third of colorectal cancer (CRC) patients develop liver metastasis (LM) within 5 years of colorectal surgery, which was originally performed with curative intent, and CRCLM is the commonest cause of death from CRC [1]. However, only one quarter of CRCLM patients are suitable for curative liver resection surgery and there is no truly effective medical therapy for either prevention or treatment of CRCLM. Therefore, there is an unmet clinical need for safe, well-tolerated therapy for prevention and treatment of metastatic CRC

There is a large body of human observational studies and experimental animal data suggesting that the omega-3 polyunsaturated fatty acids (O3FAs) C20:5 eicosapentaenoic acid (EPA) and C22:6 docosahexaenoic acid (DHA) have anti-colorectal cancer (CRC) activity [2]. Randomized clinical trial (RCT) data prove that EPA 2 g daily, in the free fatty acid (FFA) form, has chemopreventative efficacy in familial adenomatous polyposis patients [3]. Moreover, the same formulation and dose of EPA has been evaluated in patients undergoing liver resection surgery for CRCLM [4]. In this RCT (The EMT study; ClinicalTrials.gov NCT01070355), patients awaiting liver resection surgery for CRCLM were randomized to either EPA-FFA 2 g daily (n=43) or placebo (n=45) prior to surgery, for a median duration of 30 [range 12-65] days and 26 (15-73) days, respectively [4]. In this group of patients, EPA treatment was safe and well-tolerated [4]. Preliminary data suggested that pre-operative EPA treatment may improve overall survival (OS) and disease-free survival (DFS) [4].

Understanding of the mechanism(s) by which O3FAs have anti-CRC activity is limited but several putative modes of action are recognized including modulation of prostaglandin (PG) metabolism by cyclooxygenase (COX)-1 and COX-2, as well as cell surface receptor signaling [5]. A central tenet of O3FA mechanism(s) of action, pertaining to potential use in several human disease states, including cancer and cardiovascular disease, is that O3FAs exhibit anti-inflammatory properties [6].

Multiple pro-inflammatory cytokines and chemokines link inflammation and carcinogenesis, particularly in the gastrointestinal tract [7]. These include chemokine C-C motif ligand 2 (CCL2), also known as macrophage chemoattractant protein 1 (MCP-1), which has been implicated in carcinogenesis and metastasis, including in CRC and breast cancer [8, 9]. In clinical studies, treatment with O3FAs as fish oil has been demonstrated to reduce plasma and adipose tissue levels of CCL2 in obese patients with metabolic syndrome [10].

Therefore, we tested the hypothesis that EPA decreases plasma and tissue CCL2 levels in pre-clinical rodent CRC models and samples from the RCT of EPA in CRCLM patients (The EMT study).

## RESULTS

### EPA treatment is associated with decreased plasma CCL2 levels in CRCLM patients awaiting liver surgery

**Figure 1:**
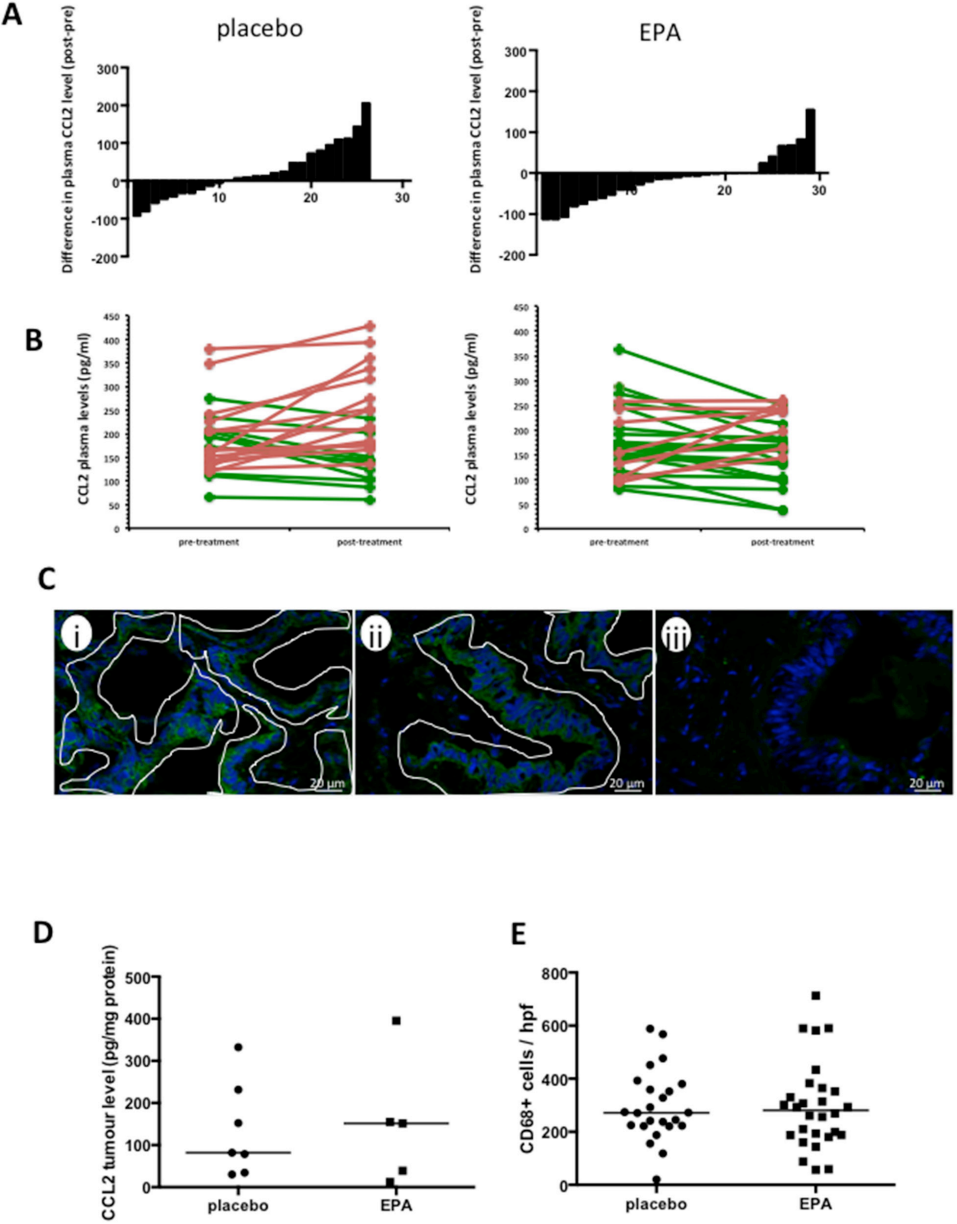
EPA treatment is associated with reduced plasma CCL2 levels in patients with CRCLMs, but no difference in CCL2 or macrophage content of CRCLMs **A.** The difference in plasma CCL2 levels (pg protein/ml) before and after intervention with placebo (n=26) or EPA (n=29), before liver resection surgery. **B.** Individual paired pre- and post-treatment CCL2 levels (increased CCL2 level after intervention, red; decreased CCL2 level after intervention, green. **C.** Localization of CCL2 in human CRCLMs by immunofluorescence. Images were obtained from patient samples with tumor tissue CCL2 levels of 225 pg/mg (i) and 34 pg/mg (ii) and plasma CCL2 levels of 135.4 pg/ml (i) and 150.8 pg/ml (ii). The fluorescence pattern indicates predominant localization of CCL2 (green) in malignant epithelial cells, (iii) negative control with omission of the primary antibody. Tumor epithelium is delineated by white lines. Stromal cells were consistently negative. **D.** Tissue CCL2 levels in CRCLMs from patients allocated to either placebo (n=7) or EPA (n=5). The bar indicates the median value for each group. **E.** CD68-positive tumor-associated macrophage content of CRCLMs from patients allocated to either placebo (n=24) or EPA (n=28). Three tumors were not analyzed as there was insufficient tissue for histological analysis. The bar indicates the median value.

As fish oil treatment for 12 weeks has been demonstrated to reduce plasma CCL2 levels in obese individuals [10], we took advantage of the prospective tissue bio-bank obtained from the EMT study [4] in order to determine whether a similar phenomenon occurred in CRC patients randomized to EPA treatment compared with placebo. Pre-treatment plasma CCL2 levels were well matched between treatment groups (P=0.66, Mann-Whitney test). However, reduction in plasma CCL2 after EPA treatment was more common (in 19 of 29 [66%] individuals) than that at the end of placebo treatment (in 10 of 26 [38%] cases; P=0.04; χ^2^ test; Figure 1A-B). Overall, there was an increase in plasma CCL2 level in the placebo group at the end of the intervention period, with a median increase in CCL2 level of +13.3 pg/ml (range −93.1 to +206.2 pg/ml), compared with an overall decrease in plasma CCL2 level in those randomized to EPA, who exhibited a median change of −12.1 pg/ml (range −119.0 to +155.1 pg/ml; P<0.05, Mann-Whitney test). These results are consistent with negative regulation of CCL2 production by EPA in patients with metastatic CRC.

### EPA decreases CCL2 synthesis by CRC cells *in vitro*

Colorectal cancer liver metastasis tissue analysis from the ‘window of opportunity’ EMT study was limited to tissue collected after intervention because tumor biopsy prior to liver surgery was not possible in patients undergoing routine CRCLM surgery [4]. Therefore, we were unable to examine whether EPA treatment reduced CCL2 content in individual CRCLMs. However, we demonstrated that CCL2 is localized predominantly to cancer cells in CRCLMs (Figure 1C) rather than in stroma-associated cells, as described previously [11]. We also measured post-treatment CRCLM CCL2 levels in a random subset of CRCLMs from the EMT trial by immunoassay. There were a wide range of CCL2 values in CRCLM tissues from patients in both placebo and EPA arms of the trial (Figure 1D). There was no statistically significant difference in tissue CCL2 content between CRCLMs from patients treated with EPA before surgery (median 151.5 [IQR 25.5-275.0] pg/mg; n=5) compared with the placebo arm (median 82.0 [IQR 34.2-231.3] pg/mg; n=7). Consistent with similar CCL2 levels in CRCLMs, there was no significant difference in the density of CD68-positive tumor-associated macrophages in CRCLMs from patients treated with placebo (median 272 cells [IQR 222-375] per hpf; n=24) compared with EPA (median 281 cells [IQR 188-362] per hpf; n=28) (Figure 1E).

**Figure 2:**
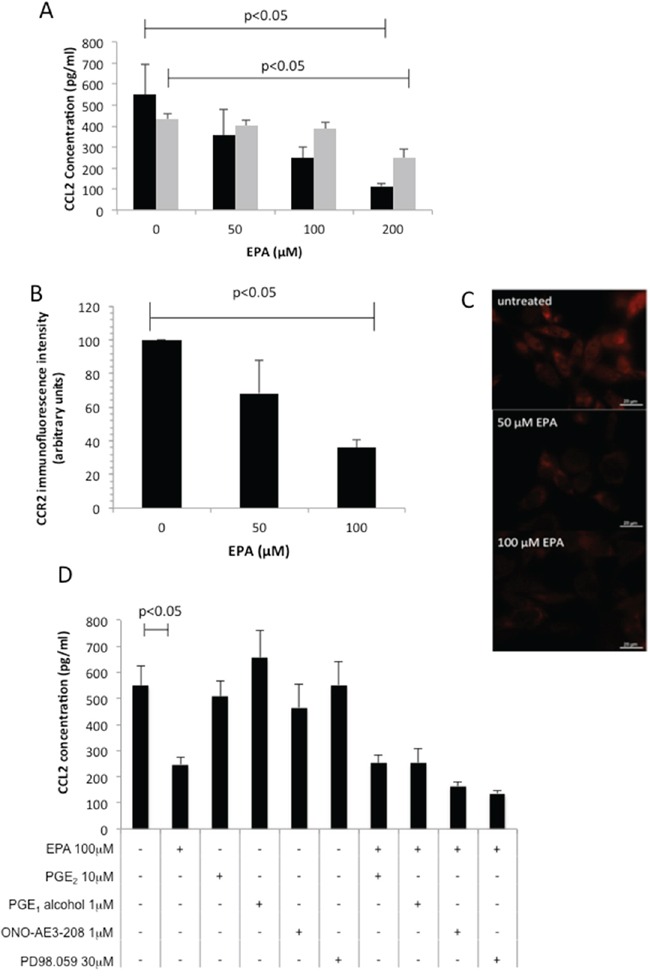
EPA inhibits CCL2 secretion and CCR2 expression in CRC cells in a concentration-dependent manner **A.** Mouse MC38 (◾) and human HCA-7 (◽) CRC cells were exposed to increasing concentrations of EPA for 24 hrs. Columns and bars represent the mean and standard deviation (n=3). **B.** CCR2 immunofluorescence intensity. Columns and bars represent the mean value and standard deviation for each condition. **C.** CCR2 protein localization in mouse MC38 CRC cells in the presence or absence of EPA for 24 hrs. **D.** Effect of EP4 receptor agonists (PGE_2_, PGE_1_-alcohol), EP4 receptor antagonist ONO-AE3-208, and the MAP Kinase inhibitor PD98059 on CCL2 production by MC38 cells. Columns and bars represent the mean and standard deviation (n=3). The Mann-Whitney test was used to test statistical significance.

We next performed a screen of three human CRC cell lines (SW480, SW620, HCA-7) and observed that only HCA-7 human CRC cells generate detectable CCL2 in conditioned medium (data not shown). There was a concentration-dependent relationship between EPA exposure and CCL2 release into medium conditioned by human HCA-7 CRC cells (Figure 2A), consistent with anti-proliferative activity of EPA against this cell line [12]. In order to further investigate the relationship between EPA treatment and CCL2 levels, we tested the effect of EPA on CCL2 production in MC38 cells, which can be used as a syngeneic CRC model in C57Bl/6 mice and are widely used to model human CRC *in vivo* based on similarities in gene expression profile [13]. Growth of MC38 mouse CRC cells was inhibited by EPA in a biphasic manner (IC_50_ values of 10.5±1.0 μM and 154.0±1.1 μM, data not shown) and was also associated with a concentration-dependent decrease in CCL2 synthesis (Figure 2A). The impact of EPA treatment on CCL2 production and cell proliferation was greater in MC38 cells than in the HCA-7 cells, which is consistent with the fact that HCA-7 cells were less sensitive to EPA than MC38 cells (HCA-7 IC_50_ 154.0±1.1 μM, data not shown). We demonstrated that EPA treatment also reduced the CCL2 receptor CCR2 expression in MC38 mouse CRC cells (Figure 2B–2C). Immunofluorescence microscopy revealed that exposure to 100 μM EPA for 24 hours decreased CCR2 expression in MC38 cells to 36±4% of the level in untreated cells (Figure 2C).

Both the CCL2-expressing CRC cell lines also exhibit constitutive COX-2 expression and PGE_2_ synthesis [12,14]. Moreover, EPA treatment inhibits PGE_2_ synthesis and downstream EP4 receptor activation in CRC cells [12]. Therefore, we investigated whether EPA decreased CCL2 production in a PGE_2_-EP4 receptor-dependent manner in MC38 cells. Neither PGE_2,_ nor the selective EP4 receptor agonist PGE_1_-alcohol, rescued down-regulation of CCL2 by EPA. In addition, neither the EP4 receptor antagonist ONO-AE3-208, nor inhibition of MAP kinase by PD98059, decreased CCL2 synthesis (Figure 2D). Therefore, EPA abrogates CCL2 synthesis and release in a PGE_2_-EP4 receptor-independent manner in MC38 mouse CRC cells.

### EPA treatment is not associated with reduced tumor CCL2 content *in vivo*

**Figure 3:**
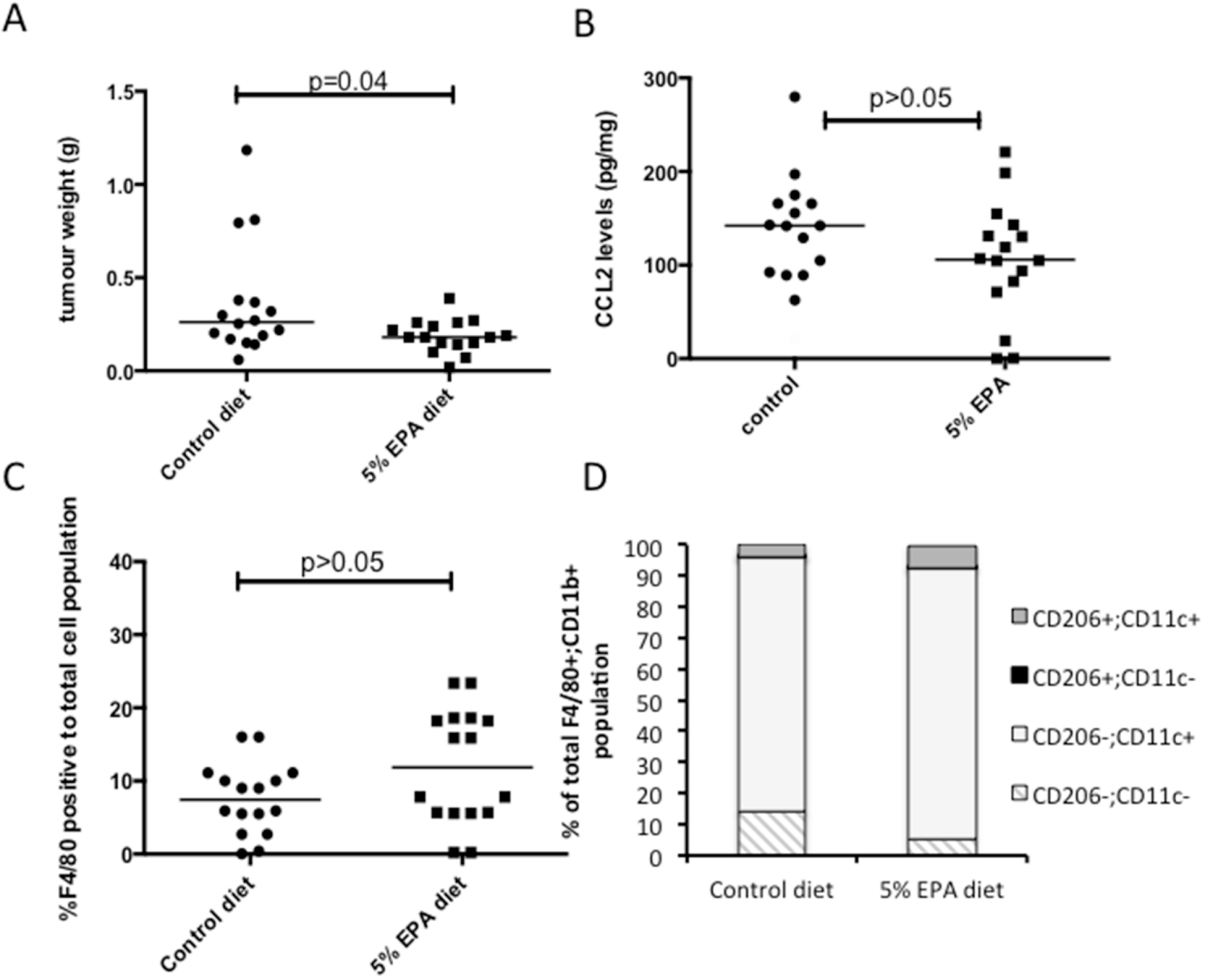
Effect of EPA on CCL2 level and tumor-associated macrophage profile in mouse MC38 CRC cell tumors *in vivo* **A.** Subcutaneous MC38 CRC cell tumor weight at sacrifice in animals provided with control or 5% EPA-containing diet (both groups n=16). **B.** CCL2 protein levels in MC38 CRC cell tumors. **C.** F4/80-positive macrophage content of MC38 CRC cell tumors. **D.** F4/80+ CD11b+ macrophage polarization analyzed by CD206 and CD11c expression.

The fact that MC38 mouse CRC cells grow readily as discrete tumors in syngeneic C57BL/6 mice allowed us to determine whether EPA treatment was associated with lower CCL2 expression *in vivo*, albeit with the similar proviso to human CRCLMs that paired pre- and post-treatment levels from the same tumor were unavailable. Animals with subcutaneous MC38 cell tumors were fed a control or 5% (w/w) EPA-containing diet. In keeping with the anti-neoplastic activity of EPA against MC38 mouse CRC cells *in vitro*, there was a significant reduction in MC38 cell tumor weight in mice provided with the 5% EPA-containing diet (median tumor wet weight 0.18 g [range 0.07-0.39 g]) compared to animals fed the control diet (0.26 g [0.06-1.19 g]; P=0.04; Figure 3A).

Control MC38 tumors exhibited a wide range of intra-tumoral CCL2 levels (median 142.0 [range 21.1-279.8] pg/mg). The levels measured in tumors from mice fed a 5% EPA-containing diet were lower (median 106.8 [0.3-220.8] pg/mg) but the difference was not statistically significant (Figure 3B), consistent with the human CRCLM data, which was also limited to post-treatment analysis. There was no significant difference in the percentage of F4/80-positive tumor-associated macrophages relative to total cell population analyzed in tumors from the two treatment groups (Figure 3C). There was also no significant difference in tumor-associated macrophage M1-M2 phenotype polarization, as determined by CD11c and CD206 phenotyping, with a dominant CD11c+ CD206- M1 F4/80+ CD11b+ macrophage population observed in both treatment groups (Figure 3D). These data suggest that anti-tumor activity of EPA in the MC38 mouse CRC model is most likely independent of tumor-associated macrophage recruitment and phenotype.

In summary, we were unable to link longitudinal reduction in plasma CCL2 following EPA treatment in humans with any significant difference in intra-tumoral CCL2 content measured *after* EPA treatment in either human or mouse tumors.

### EPA treatment does not reduce *ex vivo* CCL2 synthesis by human PBMCs

**Figure 4:**
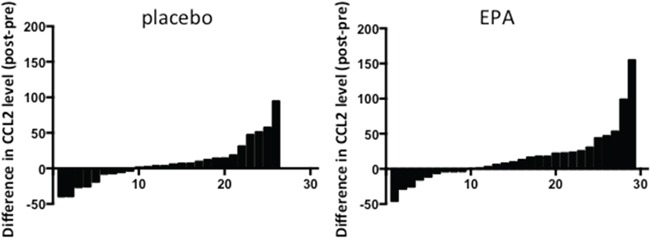
*Ex vivo* CCL2 production by LPS-stimulated PBMCs from patients treated with either placebo or EPA Bars represent the difference between post-treatment and pre-treatment levels in individual patients (placebo, n=26; EPA, n=29). Negative values indicate reduction in CCL2 level in PBMC-conditioned medium at the end of the treatment period. Conversely, positive values indicate an increase in CCL2 level in PBMC-conditioned medium after intervention.

CCL2 expression has been shown to be increased in activated human PBMCs from non-small cell lung cancer patients [15]. Therefore, we measured *ex vivo* CCL2 synthesis by LPS-treated PBMCs, which were obtained from patients in the EMT study at the same time as plasma, before and after treatment with either EPA or placebo. CCL2 protein levels in conditioned medium, normalized for PBMC number, were similar before treatment with a median CCL2 protein level of 17.9 (range 10.9-97.8) ng/ml in the control group and 28.8 (range 5.0-200.0) ng/ml in the EPA-treated group. Comparison of *ex vivo* CCL2 production by PBMCs before and after EPA or placebo did not reveal any significant difference between the groups (median differential [post- minus pre-treatment] CCL2 5.0 [range -39.9 to +94.8] ng/ml in those allocated placebo compared with 10.1 [range -462.3 to +155.3] ng/ml in the EPA treatment group; p=0.41; Figure 4). Therefore, alteration of CCL2 production by circulating monocytes is unlikely to explain the reduction in plasma CCL2 levels observed in patients treated with EPA.

### Reduction in plasma CCL2 levels after EPA treatment discriminates between different gene expression profiles in CRCLMs and predicts post-operative outcomes

**Figure 5:**
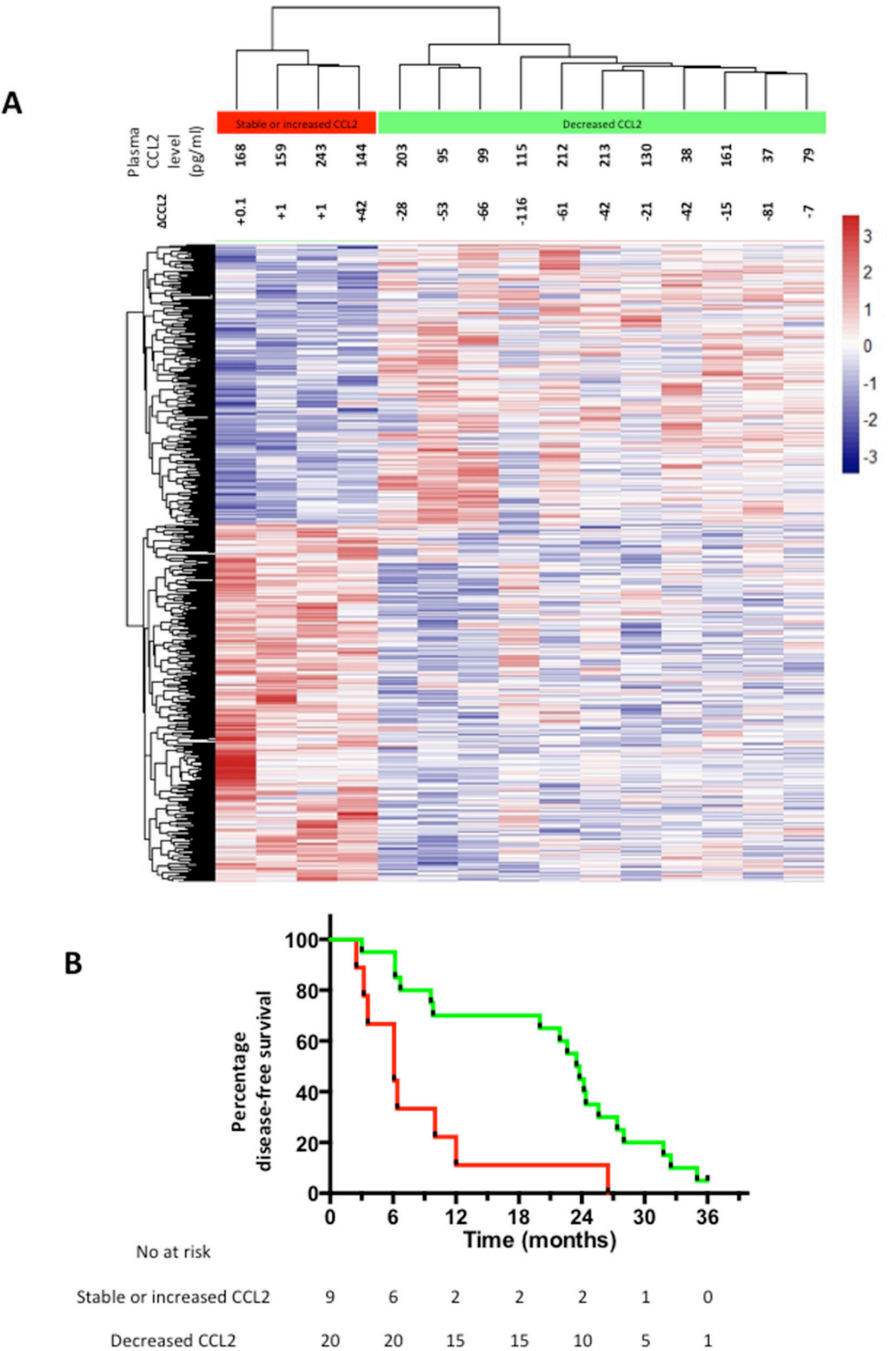
Differential gene expression in CRCLMs and clinical outcomes of EPA-treated CRCLM patients **A.** Heat map of unsupervised clustering of gene expression profiles of CRCLMs from 15 patients who received EPA, of which 4 demonstrated an increase in plasma CCL2 at the end of EPA treatment (red) and 11 demonstrated a decrease in plasma CCL2 at the end of the intervention period (green) compared with pretreatment values. The color scale indicates the range of z-score values for each sample. **B.** Kaplan-Meier curves for DFS of EPA-treated patients who either had a decrease (green line) or increase (red line) in plasma CCL2 after EPA treatment prior to CRCLM surgery. P=0.003 log rank test.

Next, we sought evidence of differences in CRCLM gene expression in EPA-treated patients who either demonstrated a reduction in plasma CCL2 levels or showed either no change or an increase in CCL2 plasma levels. Using a whole genome expression array, we identified 614 genes, which were differentially expressed (P<0.05) in tumor cell from patients with decreased CCL2 plasma levels compared to patients with no change or an increase in CCL2 plasma levels. Unsupervised hierarchical clustering of the differentially expressed genes clearly segregated CRCLMs according to the change in plasma CCL2 level after EPA treatment (Figure 5A).

MetaCore™ was used to for enrichment analysis (Supplementary Table 2). Tumors from patients who demonstrated no change or an increase in plasma CCL2 level following EPA treatment displayed enrichment of pathways linked to chemokine production and glucose metabolism, as well as down-regulation of transcription control pathways and apoptosis signaling.

A marked difference in global gene expression between CRCLMs from individuals who had either a decrease or increase in plasma CCL2 level following EPA treatment prompted analysis of post-trial clinical outcomes in these two groups.

There was a statistically significant difference in DFS after CRCLM surgery between patients whose plasma CCL2 level increased during treatment compared with individuals in whom the plasma CCL2 level decreased following treatment (Figure 5B). EPA-treated patients whose plasma CCL2 level decreased following EPA treatment had a significantly better DFS (median 23.5 months), compared with individuals in whom the plasma CCL2 level increased during EPA treatment (median 6.1 months), with a HR of 0.32 (95% confidence interval 0.05-0.51; P=0.003). By contrast, the relationship between the change in plasma CCL2 level and DFS was the opposite in the placebo arm (HR 2.09 [95% confidence interval 1.08-6.76] with a median DFS of 23.6 months and 13.8 months for patients with increased and decreased plasma CCL2 levels after placebo treatment, respectively (P=0.05). By contrast, there was no correlation between the pre-treatment plasma CCL2 level at randomization and DFS in either EMT study treatment group (data not shown).

## DISCUSSION

We conclude that treatment with the O3FA EPA is associated with an overall reduction in plasma CCL2 concentration in patients with CRCLM. Moreover, an increase in plasma CCL2 during EPA treatment predicts worse clinical outcome after CRCLM surgery and selects for a distinct CRCLM gene expression profile.

Our data are consistent with other studies, which have reported that plasma CCL2 levels in CRC patients are in the pg/ml range [16] and that O3FA treatment is associated with a reduction in circulating CCL2 concentration [10]. However, a shorter-term (8 weeks) study with daily dosing of 3.6 g mixed O3FAs did not detect a change in plasma CCL2 levels [17]. Potential confounders of the plasma CCL2 level in CRC patients include body weight and physical activity [18,19].

Tissue CCL2 content predicts poor clinical outcome in colorectal cancer and breast cancer [20–22]. Specifically, CCL2 immunoreactivity in primary CRC tumors, as measured by immunohistochemistry, predicts future liver metastasis [20] and CCL2 staining in CRCLMs predicts CRCLM recurrence and overall survival after CRCLM resection surgery [11]. However, we were unable to obtain specific, reproducible immunostaining of fixed CRCLM tissue using any commercially available anti-CCL2 antibody, with which to explore whether CRCLM content predicts survival. Circulating CCL2 levels in CRC patients have only been described once [23]. Szczepanik and colleagues have reported that the pre-operative serum CCL2 level is a prognostic factor in patients undergoing primary CRC resection [23].

We suggest that EPA treatment most likely reduces CRC cell CCL2 production in a PGE_2_-independent manner based on our *in vitro* data and those of others [24]. We also found a concomitant reduction of the CCL2 receptor CCR2 expression in CRC cells *in vitro*. This is in keeping with a study by Fang *et al*., which described a correlation between CCL2 and CCR2 expression levels in breast cancer [25]. We hypothesize that our inability to detect a reduction in tumor CCL2 content *in vivo* is due to the lack of paired human or mouse tumor tissue samples before and after EPA treatment combined with tumor heterogeneity of CCL2 expression.

It remains unclear what the cellular source of plasma CCL2 is. It is established that human colorectal adenomas and CRCs have elevated levels of CCL2 protein [11,24], which is localized to cancer cells, consistent with the production of CCL2 by human CRC cells *in vitro* [24]. We established that human CRCLMs contain CCL2 by immunoassay but were unable to detect any correlation between tissue CCL2 protein concentration and the post-treatment plasma CCL2 level in individuals from either the placebo or EPA arms of the EMT trial (data not shown). Our *ex vivo* PBMC culture experiments suggest that circulating monocytes are not the predominant source of CCL2 in plasma relevant to its role as a putative predictive biomarker, on the basis that EPA did not significantly alter *ex vivo* PBMC CCL2 production.

Surprisingly, given the role of CCL2 in tumor-associated macrophage chemotaxis [8] and association between CCL2 content and macrophage infiltrate in colonic tumors [22, 24, 26], we did not detect any effect of EPA on macrophage content of either human or mouse tumors, or any significant change in macrophage polarization in MC38 CRC cell tumors from C57BL/6 mice exposed to EPA in the diet. Despite this, it is clear that a reduction in plasma CCL2 associated with EPA treatment is linked to a marked difference in tumor biology evidenced by the differential gene expression between tumors from patients who did or did not exhibit a ‘CCL2 response’.

Importantly, the plasma ‘CCL2 response’ predicted clinical outcome in patients who received EPA treatment prior to CRCLM surgery. The predictive value of the plasma ‘CCL2 response’ to EPA treatment should be tested prospectively during planned phase III evaluation of adjuvant EPA treatment in patients undergoing CRCLM surgery. The interaction between CCL2 expression/activity and CRC recurrence requires further investigation in light of the opposite relationship between the change in plasma CCL2 level and CRC recurrence in the absence of EPA treatment, although the relatively small effect size could imply a chance finding. The possibility of combining EPA treatment with therapies targeting the CCL2/CCR2 signaling pathway should be explored. Several groups have recently reported that blocking CCL2 signaling may be effective in breast cancer and metastatic breast cancer patients [27–28]. However a recent report suggests that anti-CCL2 therapy may result in a surge in CCL2 levels and subsequent tumor growth after the treatment phase has ended [29]. It may be that targeting the CCR2 receptor will be more efficacious. A phase I trial is currently underway to assess to safety and tolerability of such an approach in pancreatic cancer patients (clinicaltrials.gov NCT02345408).

## MATERIALS AND METHODS

### Clinical samples

Blood samples were obtained from participants of the EMT study at randomization and on the day before surgery, from which peripheral blood mononuclear cells (PBMCs) and plasma samples were prepared [4]. We excluded patients with high dietary fish intake (> 2 oily fish portions per week) and/or previous users of fish oil supplements from the current analysis, providing paired samples from 55 patients (26 placebo, 29 EPA-FFA).

PBMCs were isolated from blood using Vacutainer CPT™ tubes (BD Medical, NJ, USA) and viable cells counted using a haemocytometer in the presence of 0.04% (v/v) trypan blue (Sigma-Aldrich, UK). Cells were cultured in RPMI 1640 medium (Invitrogen, UK), containing Glutamax® supplemented with 10% (v/v) heat-inactivated foetal bovine serum (FBS, Sigma-Aldrich, UK) in the presence of 1 μg/ml lipopolysaccharide (*E. coli* 026:B6; Sigma-Aldrich), for 24 hours before PBMC-conditioned medium was collected.

All samples were stored in a Human Tissue Act-approved tissue bank at -80°C. Fresh frozen tumor tissue was defrosted on ice and placed in 1 ml of ice-cold phosphate-buffered saline (PBS) supplemented with protease inhibitor cocktail (both Sigma, UK) and homogenized using a mechanical blender. Homogenates were centrifuged for 3 min at 9000 *g* to remove debris. Protein concentration was measured using a DC protein assay (Bio-Rad Laboratories Ltd., Hemel Hempstead, UK). CCL2 was measured using the R&D Systems (Abingdon, UK) human CCL2 immunoassay.

Immunohistochemistry for CD68 was performed using mouse monoclonal anti-human CD68 (KP1 clone) antibody (Dako UK Ltd., Ely, UK). Immunoreactivity was visualized using a DAKO EnVision™+ kit. CD68 scoring was performed by an operator blinded to sample allocation. Five high power field (hpf, × 200) images were acquired at random. Digital images were prepared using Leica LAS-AF Lite Software (Leica Microsystems [UK], Milton Keynes), and were further processed using Adobe Photoshop CS5.1 (Adobe Systems Inc., San Jose, CA). The mean number of stained positive cells per hpf was quantified using ImageJ software (National Institutes of Health, Bethesda, MD).

Immunocytochemistry for CCL2 was performed on OCT-embedded CRCLM tissue. Sections were thawed for 20 min at room temperature then hydrated in Tris-buffered saline (TBS) for 20 min. After blocking with Invitrogen antibody diluent (Invitrogen), sections were stained with mouse monoclonal anti-human CCL2 antibody (Millipore MABN712) followed by the AlexaFluor® 488-goat anti-mouse IgG antibody (Invitrogen). Sections were mounted using DAPI-Prolong Gold (Invitrogen) and visualized using a Zeiss AxioImager.Z1 fluorescence microscope.

### *In vitro* studies

EPA-FFA was a gift from SLA Pharma AG (Watford, UK), and is referred to henceforth as EPA. Use of EPA in *in vitro* experiments has been described previously [12]. PGE_2_, PGE_1_ alcohol and ONO-AE3-208 were obtained from Cayman (USA). PD98058 was obtained from Sigma (UK).

MC38 mouse CRC cells were a gift from D. Beauchamp (Vanderbilt University, TN, USA). HCA-7 human CRC cells were obtained from Sigma-Aldrich and RAW264.7 mouse macrophages were obtained from the ATCC (www.atcc.org). HCA-7 cells were authenticated by STR profiling. Cells were cultured in RPMI 1640 medium containing Glutamax® supplemented with 10% (v/v) FBS, at 37°C in a humidified atmosphere containing 5% CO_2_.

Cell chemosensitivity to EPA was determined using the MTT assay [30]. Briefly, cells were seeded in 96-well plates and incubated overnight at 37°C. The following day, the medium was removed and replaced with fresh medium containing EPA for 96 hrs at 37°C. Cell survival was determined using the MTT assay. IC_50_ values were calculated using Prism6™ software and are expressed as the mean ± standard deviation of three independent experiments.

Culture medium was conditioned by 1×10^5^ cells in 6-well plate wells at 37°C for 24 hrs, in the presence or absence of 50-200μM EPA, EP receptor agonists/antagonist and 30μM PD98058. CCL2 levels were measured using a mouse or human CCL2 immunoassay (both R&D Systems).

### Immunofluorescence for CCR2

Cells were seeded onto glass coverslips in 6-well plates and incubated at 37°C for 24 hrs. Cells were treated with 2 ml of fresh medium containing ethanol carrier or varying concentrations of EPA for 24 hrs. Following methanol fixation, CCR2 detection was performed using rabbit anti-mouse CCR2 antibody (GeneTex GTX61660) followed by AlexaFluor® 594 donkey anti-rabbit IgG (Invitrogen). Coverslips were mounted on slides using DAPI-Prolong Gold (Invitrogen). Fluorescence intensity was measured using ImageJ software. Integrated intensity was calculated as the mean fluorescence intensity from 5 independent fields minus the mean background intensity from 5 areas with no cellular fluorescence. Data are expressed as the mean ± standard deviation from three independent experiments

### *In vivo* studies

C57BL/6 mice were housed in a specific pathogen-free environment. All experiments were undertaken with UK Home Office approval. Female, 8 week-old C57BL/6 mice were administered isocaloric diets (n=16 per group) *ad libitum* for 14 days based on a modified AIN-93G diet base, in which 7% soybean oil was replaced by either 5% (w/w) EPA and corn oil, or corn oil alone (Supplementary Table 1). Fresh diet was manufactured by IPS Ltd. (London, UK) every 8 days and delivered within 48 hrs in vacuum-packed 100 g foil bags in order to minimize oxidation [14]. Uneaten food was replaced every day with fresh diet from a previously unopened bag.

On day 15, 1 × 10^6^ MC38 mouse CRC cells in 100 μl sterile PBS were injected subcutaneously on the flank. Animals continued on the same diet and were weighed daily for a further 14 days until sacrifice. Immediately after sacrifice, tumor weight and volume were measured blind to diet allocation. Each tumor sample was fixed in 4% (w/v) paraformaldehyde in PBS overnight, prior to embedding in paraffin, and was snap-frozen in liquid N_2_ before storage at -80°C.

Single tumor cell suspensions were prepared using 1 mg/ml type IV collagenase and 40 μg/ml DNase (Life Technologies, Paisley, UK) in HEPES-buffered saline solution (HBSS) at 37°C for 20 min. All suspensions were passed through a 4 μm cell strainer and washed thoroughly before re-suspension in PBS with 0.5% (w/v) BSA for antibody staining. Cell suspensions were stained with CD45-Peridinin Chlorophyll Protein Complex (PerCP) (30F11), CD11b-R-phycoerythrin (PE) (M1/70), CD11c-Brilliant Violet 421 (BV421) (N418) (BD Biosciences, Oxford, UK), F4/80-Fluorescein isothiocyanate (FITC) (Cl:A3-1) (AB Serotec, Kidlington, UK), CD206-Allophycocyanin (APC) (clone 15-2) (Biolegend, London, UK). Surface antigen expression was assessed by flow cytometry using a LSRII flow cytometer (BD Biosciences).

### Whole genome expression analysis

Formalin-fixed paraffin-embedded CRCLM blocks were sampled using a TMA needle as previously described [31]. Total RNA was extracted using the AllPrep DNA/RNA FFPE kit (Qiagen Ltd., Manchester, UK). Fifteen of twenty CRCLM blocks yielded total RNA of sufficient quality (RNA concentration >20 ng/μl and A_260_/A_280_ >1.95) for microarray analysis. Two samples were run with technical duplicates, which showed good reproducibility for hybridization, normalization of the data and clustering (data not shown). Whole genome expression profiles were generated using the Affymetrix GeneChip® Human Transcriptome Array 2.0 (>6 million probes). Gene expression data were pre-processed using proprietary Affymetrix® Expression Console™ v1.3 software. The raw gene expression data (CEL files) were processed using the Robust Multichip Analysis (RMA) algorithm [32] which fits a linear model at probe level allowing the effect of differences in probe-specific affinity to be minimized and providing increased sensitivity to detect small changes between experimental and control samples. The RMA algorithm performs global background adjustment of the data from each chip prior to inter-array quantile normalization. Probe level expression values were then summarized to give gene-level expression values. Raw CEL files and processed RMA files are available in the GEO repository under the series set GSE79266.

Differential gene expression analysis was performed in R [33]. The Bioconductor genefilter package was used to perform a two-sided, two-class t-test for each gene against experimental status (increased or decreased CCL2 plasma level) [34]. Genes with an equivalent fold change of ±1.2 (the median fold-change value) and a p-value of <0.05 were determined to be differentially expressed. Unsupervised hierarchical clustering of differentially expressed genes was performed using the pheatmap package [35].

MetaCore™ v6.21 (build 66768) software (Thomson Reuters, https://portal.genego.com.) was used to identify which pathways were enriched from the list of differentially expressed genes.

### Statistical analysis

The Mann-Whitney test was used to analyze data unless specified otherwise above. The log-rank test was used to assess the significance of the survival analysis exploring the relationship between changes in CCL2 levels and clinical outcomes.

## SUPPLEMENTARY FIGURES AND TABLES


