# Supplementary material for: Changes in plasma chemokine C-C motif ligand 2 levels during treatment with eicosapentaenoic acid predict outcome in patients undergoing surgery for colorectal cancer liver metastasis
Source: Oncotarget. 2016 Apr 4;7(19):28139–50. doi: 10.18632/oncotarget.8579 (PMC5053715; doi:10.18632/oncotarget.8579)
Supplement: Supplementary file 1 [file oncotarget-07-28139-s001.pdf]

Changes in plasma chemokine C-C motif ligand 2 levels during treatment with eicosapentaenoic acid predict outcome in patients undergoing surgery for colorectal cancer liver metastasis

SUPPLEMENTARY TABLES

Supplementary Table S1: Diet composition

	Control	5% EPA-FFA
Composition (% of weight)		
Protein	18.3	18.3
Fibre	5	5
Minerals and Vitamins	6.4	6.4
Carbohydrates	63.2	63.2
Fat	7.1	7.1
Corn Oil	7.0	2.0
EPA-FFA	0.0	5.0

Supplementary Table S2: Pathway enrichment analysis

Top Enriched pathways	Examples of changes in gene expression in patients with increased CCL2 plasma levels	FDR[§]	Ratio*
Transcription role of heterochromatin protein 1 family in transcriptional silencing	Downregulation of Cyclin A3, E2F1, DNMT1, Mi-1 and Tif1-beta	0.01	6/40
Apoptosis and survival. DNA-damaged induced apoptosis	Downregulation of DNA-PK, E2F1, FANCL, Histone H2AX	0.04	4/15
Phenylalanine metabolism	Downregulation of FARSA, FARSB, MIF and PheRS, Upregulation of FAAH and PAH	0.04	4/34
**Top Enriched GO Processes**			
Glucose metabolic process	Downregulation of FABP2, ATF-4, ENO, and AKT1, Upregulation of ALDOB, IBP2 and IBP3	<0.0001	22/201
Chemokine production	Downregulation of calgranulin A and B, calprotectin complex, and FOXP1	<0.0001	6/12

[§] False Discovery Rate (FDR) threshold was 0.05.
[*] Ratio of differentially expressed genes to genes involved in specific pathways or GO processes.
